# PROTOCOL: Measuring diet‐related consumer behaviours relevant to low‐ and middle‐income countries to advance food systems research: An evidence and gap map

**DOI:** 10.1002/cl2.1283

**Published:** 2022-10-11

**Authors:** Ilse de Jager, Megan Harrison, Renate F. Wit, Anne Sonneveld, Rosil Hesen, Betül T. M. Uyar, Eric O. Verger, Ana Islas Ramos, Melissa Vargas, Ramani Wijesinha‐Bettoni, Fatima Hachem, Inge D. Brouwer

**Affiliations:** ^1^ Division of Human Nutrition and Health Wageningen University and Research Wageningen The Netherlands; ^2^ Food and Nutrition Division Food and Agriculture Organization of the United Nations Rome Italy; ^3^ Wageningen Economic Research Wageningen University and Research Wageningen The Netherlands; ^4^ MoISA, Univ Montpellier, CIRAD, CIHEAM‐IAMM, INRAE, Institut Agro, IRD Montpellier France

## Abstract

This is the protocol for a evidence and gap map. The main objective of this evidence and gap map is to provide access to a systematic overview of available indicators for diet‐related consumer behaviours relevant to LMICs, to support policy makers and researchers to develop, monitor and revise food policies and programmes to leverage food systems transformations for healthier and more sustainable diets.

## 1 BACKGROUND

### 1.1 Introduction

#### 1.1.1 The problem

Today's food systems are not able to supply a nutritious, safe and affordable diet for everyone (GLOPAN, 2020). Simultaneously, food systems face many other challenges, including climate change, degradation of natural resources, population change and conflict (HLPE, 2020). There is an urgent need for a suite of effective and coherent policies and programmes to enable the transformation of food systems so they can deliver healthier and more sustainable diets, further supporting better health, environmental, and socioeconomic outcomes.

The food system is comprised of processes and actors interacting along four main components: food supply chains, food environments, individual factors and consumer behaviours (HLPE, 2020). While indicators for food production, food environments and food intake are numerous, well‐documented and (often) validated (Fanzo et al., 2021), indicators for food behaviours other than intake are limited (Kennedy et al., 2020; Melesse et al., 2020). The absence of such indicators to understand what consumers are practising, limits our ability to design, monitor and evaluate strategies aimed at supporting people's behaviours as well as to identify the aspects of the food system that contribute to them. The Food and Agriculture Organization (FAO) (FAO, 2020, p. 230) defines consumer behaviours as: ‘The actions and/or decisions made by consumers at societal, household or individual levels, on what, where and how they procure, use and dispose of food and feed others (considering gender, age and social factors); as well as actions to promote changes in their food environments. Consumer behaviours are influenced by a complex myriad of factors ranging from personal beliefs to political structures’. While still limited, research on consumer behaviour indicators is more prevalent in high‐income countries than in low‐ and middle‐income countries (LMICs) where the gap is very pronounced.

#### 1.1.2 The intervention

The aim of this study is to identify, describe and summarise indicators that have been used to measure consumer behaviours regarding the acquisition, preparation, storage, eating and disposal of food in LMICs. Given the variety of individual mediators of behaviour and the difficulty to assess their relative influence, the scope of this study will be limited to measurable food practices. For the purpose of this study, ‘practices’ were defined as behaviours that are carried out: the ‘doings’ (Schatzki, 2002). For example, the ‘frequency of households purchasing organic food by vendor type' is an indicator of a practice while the ‘proportion of households planning to buy only organic food’ is an intention. As the context (supermarket), the practitioner (a household) and the temporal dimension (frequency) are relevant parts of a practice (Wertheim‐Heck et al., 2015), these will be included.

#### 1.1.3 Why it is important to develop the evidence and gap map (EGM)

By developing an EGM, we aim to facilitate access to a systematic overview of available indicators for diet‐related consumer behaviours. A map of indicators relevant to LMICs will help advance research on food systems and support several activities such as the development, implementation, monitoring and evaluation of food‐based dietary guidelines (FBDGs). National FBDGs are a set of context‐specific and evidence‐informed multilevel recommendations to address priority dietary issues in (a) population(s), (and more recently) with a food systems approach. The EGM could therefore serve not only to help countries develop their FBDGs but also to monitor how food systems move towards healthier diets and/or more sustainable diets as recommended in national FBDGs. The indicators could serve to monitor and evaluate the effectiveness of various policy, and programme interventions to support consumer behaviours, such as food labels, marketing restrictions, controls on retail distribution and display and food taxes (e.g., sugar‐sweetened beverages tax). By doing so, those which are most successful in a particular country context can be identified and promoted.

## 2 OBJECTIVES

The main objective of this EGM is to provide access to a systematic overview of available indicators for diet‐related consumer behaviours relevant to LMICs, to support policy makers and researchers to develop, monitor and revise food policies and programmes to leverage food systems transformations for healthier and more sustainable diets. An overview of indicators will support them in identifying the most relevant indicators to collect data on, to monitor diet‐related consumer behaviours. In addition, the EGM will highlight gaps and opportunities for future development of indicators. Hence, this EGM can also help advance research on food systems in LMICs. While assessing the quality of indicators for diet‐related consumer behaviours is a critical step, it is outside the scope of this EGM.

## 3 METHODS

### 3.1 Evidence and gap map: Definition and purpose

We will undertake a systematic mapping exercise, in the form of an EGM. EGM is a new tool to support evidence‐informed policymaking. A key feature of EGMs is that they provide a visual display of evidence from systematic reviews and impact evaluations in a given sector or thematic area structured around a framework (matrix) of key interventions and outcomes (Snilstveit et al., 2016; White et al., 2020). EGMs help users to visualise the availability of existing evidence, and by providing links to user‐friendly summaries of relevant studies, EGMs can facilitate the adoption of existing evidence for decision‐making (Snilstveit et al., 2016; White et al., 2020). Since there are no existing systematic methods designed to specifically summarise indicators, we will adapt the EGM approach originally designed for effectiveness studies. A similar adaptation was recently done for the first time by Sparling et al. (2021), resulting in an interactive EGM to navigate advances in measurement in agriculture and nutrition research. Systematic reviews on other parts of the HLPE's food system framework are available, such as on the drives of food choice (Karanja et al., 2022), as well as on food environments (Turner et al. 2020) but not on consumer behaviours specifically. This study will therefore conribute methodologically and conceptually to the food system space.

### 3.2 Framework development and scope

The framework will be developed based on (1) a review of the High Level Panel of Expert's food system framework (HLPE, 2020) and other relevant papers (Kennedy et al., 2020; Melesse et al., 2020; Sparling et al., 2021), (2) the definition and conceptualisation of consumer behaviour by the FAO (FAO, 2020), and (3) consultations with experts from various disciplinary backgrounds. The scope of the EGM will include the full range of indicators measuring consumer behaviour across the entire continuum, from food acquisition to disposal.

### 3.3 Stakeholder engagement

This EGM is developed through an ongoing collaboration between the Food and Nutrition Division of the Food and Agriculture Organization of the United Nations (FAO), the ‘Global Nutrition’ Division of Human Nutrition and Health from the Wageningen University and Research (WUR) and the French National Research Institute for Sustainable Development (IRD).

FAO has conceptualised and commissioned this study and will primarily act as a technical advisory group. WUR and IRD designed the methodology, and WUR will carry out the screening and data extraction with contributions from FAO and IRD.

The whole research team met and will meet about every 4 weeks to discuss study issues, giving technical advice, inputs and feedback. In addition, the research team had and will have open ended discussions with additional experts from different disciplines relevant for this study, including the following organisations:

- Division of Human Nutrition and Health, WUR
- Wageningen Economic Research, WUR
- Health and Society, WUR
- Consumption and Healthy lifestyles, WUR
- The London Centre for Integrative Research on Agriculture and Health (LCIRAH)
- MoISA Montpellier Interdisciplinary Centre on Sustainable Agri‐food systems, IRD

### 3.4 Conceptual framework

**Figure 1:**
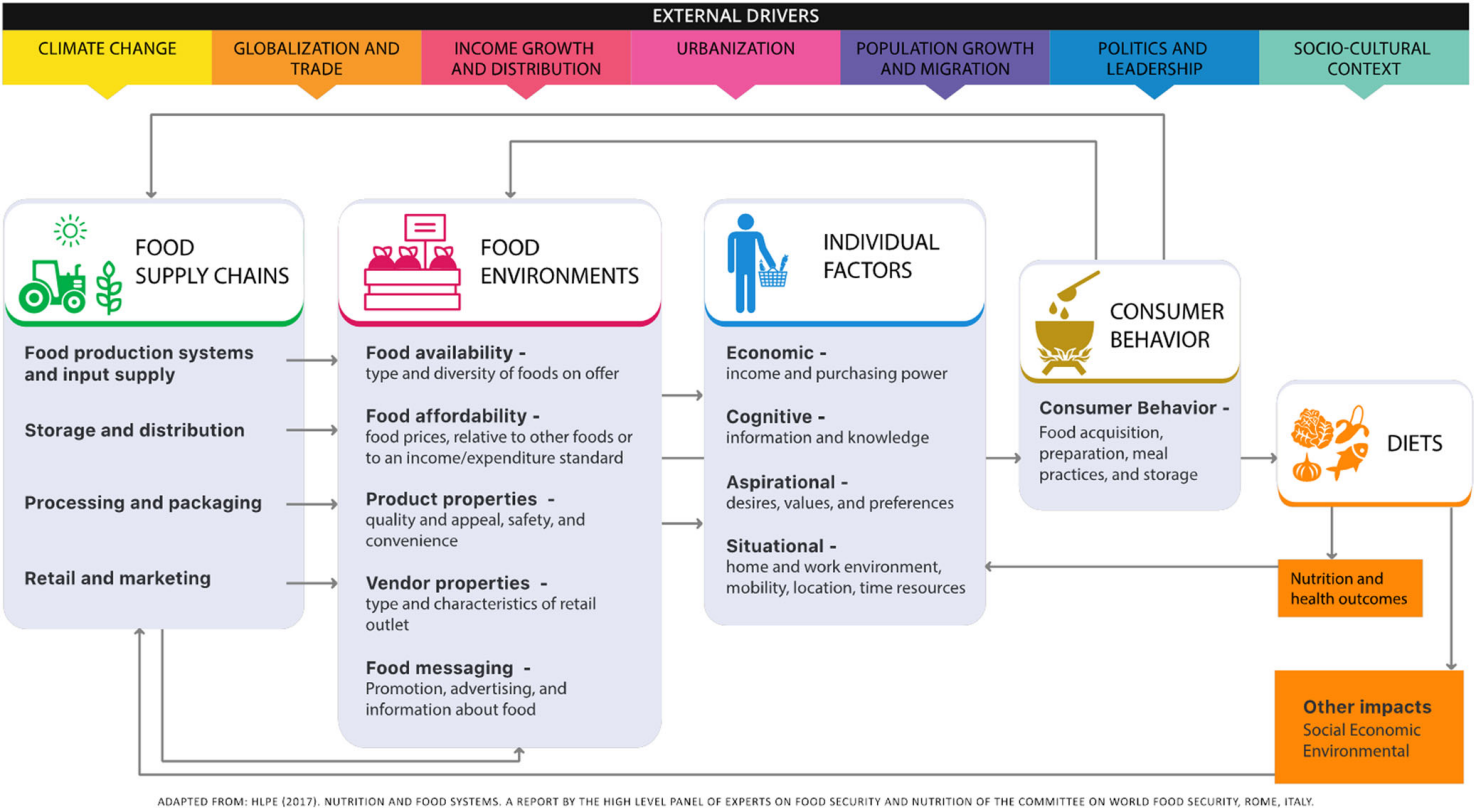
HLPE Framework, 2020 (HLPE, 2020)

The HLPE food system framework was published in 2017 as part of the report on Nutrition and food systems and includes four main components: the food supply chain, food environments, individual factors and consumer behaviour (HLPE, 2020) (see Figure 1). The recent reports by Kennedy et al. (2020) and Melesse et al. (2020) clearly show the lack of documented indicators to measure diet‐related consumer behaviours. The absence of such indicators to measure and/or describe consumer practices, limits our ability to design, monitor and evaluate strategies, along food systems, aimed at supporting people's health dietary behaviour. Hence, a complete systematic overview of consumer behaviour indicators is needed for all diet‐related domains.

### 3.5 Dimensions

The HLPE food system framework highlights the following domains for diet‐related consumer behaviours: food acquisition, preparation, meal practices, and storage (HLPE, 2020). Informed by the review of the HLPE system framework and expert consultations, we agreed on including five thematic domains that together cover a more complete range of diet‐related consumer behaviour from food acquisition to disposal. These include the acquisition, preparation, eating, storage and disposal of food. We have added the disposal of food, as this is a key issue for the environmental sustainability of food systems (HLPE, 2014). We excluded indicators for food production and for food intake within the eating domain as these are numerous, well‐documented and (often) validated. Based on the definition of consumer behaviour by the FAO (2020) and expert consultations, we also considered indicators for characterising each domain: what, where, when or how.

We organised the thatdomains around broad themes to map relevant indicators to only one domain and avoid double‐coding. Whenever more than one domain seems applicable, we will discuss and select a ‘primary’ domain and adapt the description of that domain accordingly. Regarding the type of indicator, we envision that some will measure more than one characteristic of each domain. For example, in the case of the frequency of visiting supermarkets, both where (supermarkets) and when (frequency) are measured. Accordingly, these indicators will be double‐coded. In this way, users of the EGM can easily visualize specific indicators, including those that can convey information on more than one component (what, where, when or how). As most indicators will convey information on ‘what’, we will only classify indicators as ‘what’ if they do not convey information on one of the other components. In addition, each indicator will be further categorised into subdomains (such as ‘retailer type’ within acquisition domain) so that users can more easily identify indicators for specific areas of interest. As the initial data is extracted and subdomains created, we will evaluate these subdomains using an iterative methodology.

Any indicator does not measure practices, but rather mediators (for instance, those measuring attitudes, preferences, intentions), or is not related to one of the five domains will be excluded. Ineligible indicators from included papers will not be included in the EGM, while all of the eligible indicators will be included. Papers may include multiple indicators, with each being extracted separately. The following data elements will be extracted for each indicator: target group level (individual, household, community; different age groups; and gender), scope (global, national, regional, district, city, village and neighbourhood), location of study, and other cross‐cutting aspects (such as economy, food safety, gender, water and hygiene, sustainability).

Similarly, to Sparling et al. (2021), rather than displaying interventions as rows in the matrix of the EGM map, we will include the five thematic domains. Categories of indicators will be included in the columns of the matrix of the EGM, instead of the study outcomes as seen in traditional effectiveness maps. Table 1 includes descriptions of each domain, including examples of indicators.

**Table 1:** Domains covering the full range of diet‐related consumer behaviours including the different types of indicators within each domain

Domain	**Description** (including examples of the different types of indicators measuring what, where, how or when)
Acquisition	The acquisition of food involves all the actions related to obtaining food for consumption.
	***what*** types of food consumers acquire (types of foods at home, average amount of type of food (group) purchased, total household food expenditure); ***where*** consumers acquire food and/or a meal (share from own production, wild and markets); ***how*** a consumers acquire food (mode of payment, transportation mode); ***when*** consumers acquire food (frequency of acquisition, types of food/season, time required for food shopping).
Preparation	Food preparation involves the actions that are performed to prepare food to ensure that the food consumed is safe to eat, safe to store, to enhance sensory characteristics, and/or to follow personal and/or cultural preferences.
	***what*** types of food consumers prepare (types of food prepared, amount of type of food prepared) ***where*** food is prepared (number of small food stalls in an area, availability and cleanness of the kitchen, number of meals prepared at home), ***how*** a food is prepared (food preparation methods, food preparation skills, use of safe portable water, use of a mill, availability of cooking equipment, access to electricity for cooking, hygiene practices such as using same chopping board to prepare raw and cooked foods or cooking time for meat or fish) and ***when*** food is prepared during the day and for what moment (frequency of preparation, time required for food preparation: share of population accessing water in more than 30 min, average time required for cooking: share of population with access to clean fuels and technologies for cooking).
Eating	The act of consuming food.
	***what*** types of foods are consumed (type of food groups consumed, types of food groups not consumed), ***where*** food/meals are consumed (share of consumed food in and outside of home, in front of the TV), ***how*** a food/meals are consumed (meal allocation: who makes this choice, WEAI, plate sharing, eating alone or in company) and ***when*** food/meals are consumed (frequency of eating, meal frequency, skipping meals, time dedicated to meals, seasonal differences for types of foods and/or the number of meals per day, festivities).
Storage	Food storage includes all actions that allow both cooked and raw materials to be eaten for some time after harvest (enabling food distribution, a balanced diet throughout the year, and reducing food waste).
	***what*** types of foods are stored (types of foods stored at home and in food stalls), ***where*** food is stored (dry storage, availability of refrigerated or frozen storage), ***how*** a food is stored (storage at room temperature, drying/salting, raw and cooked foods close together in the refrigerator) and ***when*** food is stored (frequency of storage, which foods in which season and when stored food is used).
Disposal	The actions involved to get rid of the food that is not eaten, and the packaging of food: the collection, processing and recycling or deposition of food waste.
	***what*** (part of) foods are disposed (types of food disposed, percentage of food lost at home, average amount of household food wasted), ***where*** foods are disposed (share of population that uses practises that contaminate portable water supply or other foods), ***how*** a foods are disposed (percentage of total food waste that is recycled, time required for cleaning up), and ***when*** foods are disposed (frequency of disposal, common disposal practices per season)

^a^ An indicator will only be categorised as ‘how’ when the indicator specifically describes details of how a specific practice is done: the action verb in the indicator is followed by a description of how that action was carried out (e.g., Proportion of households thawing raw chicken by leaving it at room temperature).

**Table 2:** Heatmap of the number of indicators by thematic domain (rows), against types of indicator (columns)

Domains	Type of indicators			
	What	Where	How	When
Acquisition	# of indicators	# of indicators	# of indicators	# of indicators
Preparation	# of indicators	# of indicators	# of indicators	# of indicators
Eating	# of indicators	# of indicators	# of indicators	# of indicators
Storage	# of indicators	# of indicators	# of indicators	# of indicators
Disposal	# of indicators	# of indicators	# of indicators	# of indicators

**Table 3:** Format of the interactive evidence and gap map showing thematic domains and subdomains (rows), against type of indicators and individual indicators (columns)

			Indicators							
			What		Where		How		When	
			Indicator	Indicator	Indicator	Indicator	Indicator	Indicator	Indicator	Indicator
**Domains**	Aquisition	Subdomain								
		Subdomain								
	Preparation	Subdomain								
		Subdomain								
	Eating	Subdomain								
		Subdomain								
	Storage	Subdomain								
		Subdomain								
	Disposal	Subdomain								
		Subdomain								

*Note*: Options to filter for: population, country of study, scope, critical appraisal and other cross‐cutting aspects.

#### 3.5.1 Types of study design

Primary research of any design (well‐established study designs such as cross‐sectional studies and experimental studies, new designs, validation study) and which are fully published will be included. Reviews will be excluded for data extraction but recorded elsewhere to help with the analysis and discussion. We do not plan to include qualitative research.

#### 3.5.2 Types of intervention/problem

Table 1 includes descriptions of each thematic domain relevant to diet‐related consumer behaviours (analogous to the traditional use of ‘interventions’ in most EGMs), including examples of potential indicators of different types (analogous to ‘outcomes’).

#### 3.5.3 Types of population (as applicable)

Only studies which collect data in, or develop indicators for LMICs, as defined by the World Bank, will be included. We will exclude nongeneralisable population groups with specific nutritional needs (e.g., people with asthma, celiac disease or athletes). But we will include people with infections (e.g., HIV and malaria), people who are malnourished, pregnant and lactating women, because these groups are common in LMICs.

#### 3.5.4 Types of outcome measures (as applicable)

In this study the primary outcomes are indicators. Only quantitative or semi‐quantitative (indicators based on questionnaires that ask for quantitative answers but are not measured, for example, reported travel time to the place someone buys most of his or her food) indicators will be included.

#### 3.5.5 Other eligibility criteria

Other inclusion criteria are:

- Developed and published during the last 10 years (2011–2021)
- In any country categorised as low income, low‐middle income or middle‐high income by the World Bank in 2021
- Primary research of any design (well‐established study designs, new designs, validation study, users guide for a new indicator)
- In published literature
- Include quantitative and semi‐quantitative indicators (estimation of the quantity)
- Peer‐reviewed journal articles with full‐texts in English, Spanish or French

Other exclusion criteria are:

- Indicators related to individual determinants of behaviour (e.g., attitudes, knowledge, intentions).
- Indicators related to growing food/food production
- Indicators related to dietary intake
- Studies of niche or nongeneralisable populations (hospital patients, athletes)
- Indicators targeted specifically at infants and young children

### 3.6 Search methods and sources

We will employ a comprehensive published literature search of two databases, Web of Science and Scopus with search terms (listed in Supporting Information: Appendix 2) and we will keep track according to the PRISMA principles. We will choose the 10‐year period based on the assumption that the most used indicators will be reflected.

### 3.7 Analysis and presentation

#### 3.7.1 Report structure

The report will include the following tables and figures may include:

- PRISMA diagram
- A visual presentation of the EGM (heat map listing the number of indicators per cell (indicating both the availability of indicators per domain and type of indicator), see Table 2
- A link to the interactive EGM (which will include all individual indicators, as well as subdomains), see Table 3
- A visual and numeric summary of domains with most indicators (chord diagram, proportion of studies per indicator, per measurement level, per domain)

#### 3.7.2 Filters for presentation

Additional filters will be added to the interactive map, including: subtypes of indicators within each domain and type of indicator, target group levels (by unit [individual, household, community], by age [children, adolescents, adults, older adults]), by gender (only female, only male, mixed [i.e., both male and female]); geographical scope (national, subnational, regional, district, city, village, neighbourhood); and other cross‐cutting domains (economy, food safety, gender, water and hygiene, sustainability).

#### 3.7.3 Dependency

The unit of analysis will be at the indicator level. One study might have used more than one eligible indicator. In this case, all the indicators will be added separately to the EGM with reference to the same study. This means that one study maybe included multiple times in the EGM. In addition, the same indicator can be used in different studies. In this case, the indicator will be included once in the EGM but with reference to two or more studies. This means that the number of indicators included in the EGM will likely be greater than the number of studies included.

### 3.8 Data collection and analysis

#### 3.8.1 Screening and study selection

Three independent experiencedresearchers (Anne Sonneveld, Renate Wit and Ilse de Jager) will search and then double screen the first 10% of the search results on title and abstract, with a fourth researcher (Inge Brouwer) providing a decision in the case of disagreement. For the remaining search results, the three independent researchers will single screen title and abstract and discuss all issues in weekly meetings. In case of disagreements during these meetings, the fourth researcher will make a decision. The full‐text screening will be performed by five independent researchers (Rosil Hesen, Anne Sonneveld, Renate Wit, Megan Harrison and Ilse de Jager), all articles will be double screened with a sixth researcher (Inge Brouwer or Eric Verger) providing a decision in the case of disagreement. Double screening of title and abstract and full text of articles in Spanish and French will be carried out by three researchers (Melissa Vargas, Ana Islas Ramos and Eric Verger) and a fourth researcher (Inge Brouwer or Ilse de Jager) will provide a decision in case of disagreement.

The team of researchers will screen titles and abstracts according to the inclusion and exclusion criteria, and further screen full‐text publications, using the software CADIMA. The screening criteria questions will be clear and concise, objective, ‘single‐barrelled’, same sentence structure, with only yes/no answers, and easiest questions at the beginning (Polanin et al., 2019). Multiple consistency checks will be done until the team reaches consensus. As advised for large‐evidence reviews, the team will meet on a weekly basis to avoid individual decisions that differ from the group's decision‐making process (Polanin et al., 2019).

#### 3.8.2 Data extraction and management

Five independent researchers (Rosil Hesen, Renate Wit, Megan Harrison, Betül Uyar and Ilse de Jager) will code papers using EPPI Reviewer 4. We will collaboratively extract the data, including cross‐checking the data extracted from each paper and having weekly discussions with the team. An Excel sheet will be used to keep track who reviewed which paper as well as related questions regarding the data extraction. A second reviewer will look at the same paper and answer questions of first reviewer. If questions/disagreements still remain, these will be discussed during the group meetings. In case disagreements remain after cross‐checking and discussions, two other researchers (Inge Brouwer and Eric Verger) will provide a decision. Melissa Vargas, Ana Islas Ramos and Eric Verger will extract the data of the articles in Spanish and French with disagreements resolved by Inge Brouwer and Ilse de Jager. We do not plan to use any automation or text‐mining.

Besides the rows of ‘intervention’ (domains) and columns of ‘outcomes’ (types of indicators) described previously, we propose to code for several other factors that will act as filters in the EGM. Some filters will have predefined categories:

- target group levels, by unit
  o individual/household/community
- target group levels, by age
  o children (<12 years)/adolescents (12–18 years)/adults (>18 years)/older adults (>65 years)
- target group levels, by gender
  o only female/only male/mixed (i.e., both male and female)
- geographical scope
  o national/subnational/regional/district/city/village/neighbourhood

Some filters will not have predefined categories:

- Subdomains (e.g. ‘retailer type’ within acquisition domain and where type of indicators)
- Country/countries of study
- Other cross‐cutting aspects (economy, food safety, gender, water and hygiene, sustainability)

The full coding sheet that will be pre‐tested is included in Supporting Information: Appendix 3. We will compose a database listing all indicators and coded data extracted for each indicator using EPPI Mapper (an interactive EGM).

#### 3.8.3 Tools for assessing risk of bias/study quality of included reviews

Instead of a risk of bias assessment in effectiveness studies, we will assess the quality of the indicators by several parameters based on validity literature, including:

- Data collecting tool accessibility (whether the tool used, e.g., the questionnaire, is available or referred to)
  o yes/no
- Data quality (whether the data collected for the indicator is self‐reported, observed or a mix of both)
  o self‐reported data/observed data/both self‐reported and observed data
- Data‐driven index (if the indicator has an index, whether the index is based on the data of the study population itself, reflecting generalisability)
  o yes/no (if no index also code as no)
- Open access (whether the paper is open access)
  o yes/no
- Data accessibility (whether the data needed for the indicator is publicly available, e.g. in Living Standard Measurement Studies (LSMS) by the World Bank)
  o yes/no

In addition, the level of adoption of each indicator will be indicated by the frequency count a single indicator was used across different studies (shown in interactive EGM).

#### 3.8.4 Methods for mapping

For the development of the EGM, we will use the software CADIMA for screening the literature, EPPI reviewer 4 for data extraction and EPPI mapper for producing the visual interactive EGM.

## SOURCES OF SUPPORT

**Internal sources**

- New Source of support, Other

**External sources**

- No sources of support provided

## Supporting information

Supporting information.
