# Supplementary material for: PROTOCOL: Measuring diet‐related consumer behaviours relevant to low‐ and middle‐income countries to advance food systems research: An evidence and gap map
Source: Campbell Syst Rev. 2022 Oct 11;18(4):e1283. doi: 10.1002/cl2.1283 (PMC9551704; doi:10.1002/cl2.1283)
Supplement: Supplementary file 1 — Supporting information. [file CL2-18-e1283-s001.docx]

# Appendices

## 1 Link to online interactive EGM

Add link at the full report stage

## 2 Search strings

The literature search was performed on the 28th of April 2021 in two databases: Web of Sciences (WoS) and Scopus.

The search string consisted of various parts as described in the tables below.

(A AND (B within 3 words from C) AND (1 OR 2 OR 3 OR 4 OR 5)) AND NOT (I OR II OR III). In addition, criteria were set to IV and V.

|  | A | B | C |
| --- | --- | --- | --- |
| **Concept** | **Food** | **Consumers** | **Behaviour** |
| **Search strings** | Food*  Meal* | Consumer*  Customer*  Household*  Women | Behavio*  Practic* |

|  | 1 | 2 | 3 | 4 | 5 |
| --- | --- | --- | --- | --- | --- |
| **Concept** | **Acquisition** | **Preparation** | **Storage** | **Eating** | **Disposal** |
| **Search strings** | Buy*  Shop*  Purchas*  Obtain* | Prepar*  Proces*  Cook*  Heat*  Warm*  Simmer*  Boil*  Steam*  Bak*  Roast*  Fry*  “deep fry*’’  Clean* | Stor*  Preserv*  Sav*  Cover*  Refrigerat*  Cool*  Bag  Graner*  Salt*  Dry*  Pack* | Eat*  Consum* | Dispos*  Wast*  Recycl*  Throw*  Dissipat* |

|  | I | II | III | IV | V |
| --- | --- | --- | --- | --- | --- |
| **Other criteria** | “United States”  UK  “United Kingdom”  USA  Australia*  Europe  European*  Canada  Canadian*  Germany  Infant*  “Infants and young children” | WOS articles excluded  from category:    Veterinary sciences | Scopus  articles excluded from category:    Chemistry    Veterinary sciences | Year published:    After 2010 | Language limited to:    English    Spanish    French |

Below are the searches for the ENGLISH articles. Please mind that for the Spanish and French articles the same search string was used, except that the language was changed.

In Web of Science, 3162 articles were found using the following search string:

(TS=((Food* OR Meal*) AND ((consumer* OR customer* OR household* OR women) NEAR/3 (behavio* OR practic*)) AND ((buy* OR shop* OR purchas* OR obtain*) OR (eat* OR consum*) OR (stor* OR preserv* OR sav* OR cover* OR regfrigerat* OR cool* OR bag OR graner* OR salt* OR dry* or pack*) OR (prepar* OR proces* OR cook* OR heat* OR warm* OR simmer* OR boil* OR steam* OR bak* OR roast* OR fry* OR "deep fry*" OR clean*) OR (dispos* OR wast* OR recycl* OR throw* OR dissipat*))) NOT TS=(("united states" OR UK OR "united kingdom" OR USA OR australia* OR europe OR european* OR canada OR canadian* OR germany OR infant* OR "infants and young children"))) AND ((PY==("2021" OR "2020" OR "2019" OR "2018" OR "2017" OR "2016" OR "2015" OR "2014" OR "2013" OR "2012" OR "2011") AND LA==("ENGLISH")) NOT (TASCA==("VETERINARY SCIENCES")))

In Scopus, 4943 articles were found using the following search string:

((TITLE-ABS-KEY((food* OR meal*) AND (consumer* OR customer* OR household* OR women W/3 behavio* OR practic*) AND (buy* OR shop* OR purchas* OR obtain*)) AND NOT TITLE-ABS-KEY("united states" OR UK OR "united kingdom" OR USA OR europe OR european* OR canada OR canadian* OR germany)) AND PUBYEAR > 2010) OR ((TITLE-ABS-KEY((food* OR meal*) AND (consumer* OR customer* OR household* OR women W/3 behavio* OR practic*) AND (eat* OR consum*)) AND NOT TITLE-ABS-KEY("united states" OR UK OR "united kingdom" OR USA OR australia* OR europe OR european* OR canada OR canadian* OR germany)) AND PUBYEAR > 2010) OR ((TITLE-ABS-KEY((food* OR meal*) AND (consumer* OR customer* OR household* OR women W/3 behavio* OR practic*) AND (stor* OR preserv* OR sav* OR cover* OR regfrigerat* OR cool* OR bag OR graner* OR salt* OR dry* or pack*)) AND NOT TITLE-ABS-KEY("united states" OR UK OR "united kingdom" OR USA OR australia* OR europe OR european* OR canada OR canadian* OR germany)) AND PUBYEAR > 2010) OR ((TITLE-ABS-KEY((food* OR meal*) AND (consumer* OR customer* OR household* OR women W/3 behavio* OR practic*) AND (prepar* OR proces* OR cook* OR heat* OR warm* OR simmer* OR boil* OR steam* OR bak* OR roast* OR fry* OR "deep fry*" OR clean*)) AND NOT TITLE-ABS-KEY("united states" OR UK OR "united kingdom" OR USA OR australia* OR europe OR european* OR canada OR canadian* OR germany)) AND PUBYEAR > 2010) OR ((TITLE-ABS-KEY((food* OR meal*) AND (consumer* OR customer* OR household* OR women W/3 behavio* OR practic*) AND (dispos* OR wast* OR recycl* OR throw* OR dissipat*)) AND NOT TITLE-ABS-KEY("united states" OR UK OR "united kingdom" OR USA OR australia* OR europe OR european* OR canada OR canadian* OR germany)) AND PUBYEAR > 2010) AND NOT (infant* OR "infants and young children") AND ( EXCLUDE ( SUBJAREA,"CHEM" ) OR EXCLUDE ( SUBJAREA,"VETE" ) ) AND ( LIMIT-TO ( LANGUAGE,"English" ) OR EXCLUDE ( LANGUAGE,"Chinese" ) OR EXCLUDE ( LANGUAGE,"Korean" ) OR EXCLUDE ( LANGUAGE,"Russian" ) OR EXCLUDE ( LANGUAGE,"Polish" ) OR EXCLUDE ( LANGUAGE,"Italian" ) OR EXCLUDE ( LANGUAGE,"Japanese" ) OR EXCLUDE ( LANGUAGE,"Croatian" ) OR EXCLUDE ( LANGUAGE,"Hungarian" ) OR EXCLUDE ( LANGUAGE,"Turkish" ) OR EXCLUDE ( LANGUAGE,"Persian" ) OR EXCLUDE ( LANGUAGE,"Romanian" ) OR EXCLUDE ( LANGUAGE,"Ukrainian" ) OR EXCLUDE ( LANGUAGE,"Afrikaans" ) OR EXCLUDE ( LANGUAGE,"Czech" ) OR EXCLUDE ( LANGUAGE,"Moldavian" ) OR EXCLUDE ( LANGUAGE,"Moldovan" ))

## 3 Data extraction form

1. Localize potentially relevant documents with search terms
2. Re-evaluate search terms and/or inclusion and exclusion criteria according to search results
3. Repeat step 1 and 2 until satisfied with search results
4. Download documents and store in repository (using CADIMA software)
5. A code, full title, link to full report will be uploaded in CADIMA
6. Start title and abstract screening, using the following criteria:
   1. Does the study population consist of humans (niche population: no)?
   2. Does the study include low- and middle-income context? (not sure/not specified: yes)?
   3. Is the study about food or food groups (specific food item: no)?
   4. Is the scope of the study related to at least one of the following domains: acquisition, preparation, storage, eating (excluding food intake), or disposal of food?
   5. Does the abstract of the study refer to practices?
7. Double screen first 10%, discuss inconsistencies
8. Single screen other 90%, keep track of uncertainties in shared Excel, discuss during weekly meetings
9. Start full-text screening, using the following criteria:
   1. Is the study primary research?
   2. Does the study include low- and middle-income context?
   3. Does the study include practices related to one of the following domains: acquisition, preparation, storage, eating (excluding food intake), or disposal of food?
   4. Does the study include indicators for practices?
10. Double screen all papers, discuss inconsistencies
11. Upload selected papers in EPPI reviewer 4
12. Trial rounds for coding and using EPPI mapper
13. For each eligible indicator in each screened document, organise according to **thematic domain**.
    1. Acquisition
    2. Preparation
    3. Cooking
    4. Storage
    5. Eating
    6. Disposal
14. For each eligible indicator in each screened document, organise according to **type of indicator**.
    1. (only) What
    2. Where
    3. How
    4. When
15. Add short description of the indicator
16. For each eligible indicator in each screened document, organise according to **subtype of indicator**(these will be defined during the coding process, e.g. fast food restaurants).
17. For each eligible indicator in each screened document, add **country/countries of study**
18. For each eligible indicator in each screened document, add information on **population**:
    1. Type of population
       1. Individuals
       2. Households
       3. Communities
    2. Age
       1. Children (4-11y)
       2. Adolescents (12-17y)
       3. Adults (18-65y)
       4. Older adults (65+)
       5. Households
    3. Gender
       1. Only female
       2. Only male
       3. Mixed
19. For each eligible indicator in each screened document, add **geographical scope**(select lowest level traceable)**:**
    1. Global
    2. National
    3. Regional
    4. District
    5. City
    6. Village
    7. Neighbourhood
20. For each eligible indicator in each screened document, select applicable **other crosscutting domains**(add others during coding process if needed):
    1. Economy
    2. Food safety
    3. Gender
    4. Sustainability
    5. Water, sanitation and hygiene
21. For each eligible indicator in each screened document, execute critical appraisal:
    1. Data collection tool accessible
       1. yes
       2. no
    2. Data driven index (generalisability)
       1. yes
       2. no
    3. Article open access
       1. yes
       2. no
    4. Data public accessible (to add later after coding, need to check databases)
       1. yes
       2. no
22. Cross-check all data extracted, keep track in Excel, discuss during weekly meetings
23. Create EGM using EPPI mapper
